# Optimizing biologic treatment in IBD: objective measures, but when, how and how often?

**DOI:** 10.1186/s12876-015-0408-x

**Published:** 2015-12-18

**Authors:** Shomron Ben-Horin, Ren Mao, Minhu Chen

**Affiliations:** IBD service, Department of Gastroenterology, Sheba Medical Center & Sackler School of Medicine, Tel-Aviv University, Tel Hashomer, 52621 Israel; Department of Gastroenterology, First Affilated Hospital, Sun-Yatsen University, 58 Zhongshan II Road, Guangzhou, 510080 P.R. China

## Abstract

**Background:**

The advent of biologic agents for the treatment of inflammatory bowel disease (IBD) was accompanied in parallel with emerging understanding of persisting underlying inflammation and ensuing bowel damage that can occur even in patients with seeming clinical remission. This lead to the concepts of mucosal healing and deep remission gaining acceptance as the more desired goals for therapy within an ambitious disease-control therapeutic approach, namely, treat-to-target strategy. However, how to practically monitor IBD patients, which objective measures to follow, at what time-points and whether to act upon results in asymptomatic patients are all questions that remain disputed.

**Methods and result:**

In this concise review we aim to provide an overview of objective measures for monitoring of IBD patients, focusing on the challenging group of patients treated by infliximab, adalimumab, vedolizumab and other biologics. These objective measures are discussed in the context of the different common clinical scenarios wherein the clinician may contemplate their use. Specifically, we will delineate the role of objective parameters to be monitored during induction phase of treatment, during maintenance therapy, at loss of response and after elective cessation of therapy in patients in remission.

**Conclusion:**

Coupled with the non-negligible costs of therapy, and the over-all worse prognosis of moderate-severe patients who are the usual recipients of biologic therapies, this challenging patients seem to be the first candidates for this more proactive strategy combining inflammatory and pharmacokinetic monitoring of objective inflammatory and pharmacokinetic measures. More data is still desirable to better define the exact parameters to be followed and their optimal thresholds, and to delineate the optimal cost-effective interventions for these patients.

## Background

The advent of novel biologics for the therapy of inflammatory bowel disease (IBD) has substantially increased the ability to induce and maintain remission of patients. With clinical response and remission increasingly being achieved, attention has spun to more ambitious goals, specifically aiming at altering the natural debilitating course of these diseases. This lead to investigations of objective markers, to be set as surrogate goals for therapy thereby increasing the probability of long-term favourable outcome. Taken together with the considerable costs of biologics, interest in monitoring the therapy has risen not only for the purpose of identifying markers that will serve as end-points for successful treatment, but also for timely cessation or switching of therapy in those unlikely to respond. In this concise review we will examine emerging markers and strategies, on which rational optimization of therapy of IBD may be achieved with a focus on the optimization of biologic therapies in different stages of treatment.

### Before and during the induction phase of treatment

The single most important factor for optimization of therapy for IBD at the initiation of treatment is ascertainment of active IBD inflammation. While this may seem trivial, one often finds various circumstances in clinical practice lead to commencement of therapy without clear-cut indicators of active inflammation and without rigorous exclusion of other aetiologies for symptoms. These management ‘short-cuts’ will ultimately lead to greater number of patients being treated with immune-modulators and/or biologics for symptoms arising from causes other than active inflammation, subsequently increasing therapeutic failure rate. For instance, in a real life cohort of 438 Crohn’s disease (CD) patients, elevated CRP was associated with three-time higher likelihood to respond to adalimumab induction [1]. Similar results were obtained with infliximab from both real-life cohorts [2] and from sub-analysis of ACCENT I infliximab Trial, whereby elevated CRP at baseline was associated with better response to induction and durable response [3]. However, it is notable that depending on the outcome assessed, elevated baseline CRP may also be associated with worse clinical outcome. Thus, when a rigorous and severe outcome in the form of future colectomy was assessed among infliximab treated UC patients, high baseline CRP rather than a normal one predicted higher infliximab failure as gauged by subsequent need for colectomy [4]. This probably reflects the fact that the high-CRP population is enriched with genuine inflammatory patients rather than diluted by some IBD-IBS or by mild UC patients with favourable prognosis regardless of therapy. It follows that evidence of baseline inflammation should be correctly appreciated as a double-edge sword. Thus, several studies showed higher medical therapy failure rates in patients with very high level of CRP or with severe endoscopic lesions, either among patients with acute severe colitis [5] or with CD [6] receiving induction infliximab treatment. One interpretation of these data may be to despair from medical therapy and opt for early referral for surgical therapy, which may be justified approach in some patients. However, an alternative interpretation may be that these patients with high inflammatory indices are afflicted with severe inflammatory burden which will require more intensive medical therapy. Indirect evidence that this may be the case was provided by sub-analysis CHARM trial pertaining to the response to weekly versus bi-weekly adalimumab therapy for high versus low CRP groups [7]. Similarly, a retrospective analysis indicated an accelerated induction with Infliximab may be superior to conventional induction in patients with acute severe colitis when compared to a historic cohort [8], although no direct correlation with underlying inflammatory burden grades was performed. Nonetheless, whether pharmacokinetics in the form of inadequate drug-exposure is the cause for most induction failures has been challenged by a recent elegant study from Germany. The investigators showed by fluorescent-conjugated adalimumab staining of mucosa during confocal endoscopic microscopy that most patients who did not respond to adalimumab had low burden of TNF+ mucosal cells [9]. These findings imply that most primary failures of anti-TNF are due to inflammation propagated by non-TNF mediated pathways, rather than by overwhelming TNF burden that may justify an increase in the induction dose. Indeed, there is conflicting pharmacokinetic data regarding the usefulness of lower infliximab levels at week 2 as predictors of subsequently failing versus responding patients [10, 11].

Interestingly, while vedolizumab treated UC patients with high-end quartile drug concentrations seemed to enjoy higher rate of clinical response and remission at week 6 [12], this is a somewhat a bewildering observation given the fact that even lower doses than tested in the trial achieve near 100 % binding-saturation of the alpha4beta7 target integrin [13]. Moreover, no clear correlation was demonstrated between base-line inflammation in the form of calprotectin values above 500mcg/gr, and the rate of response to therapy at week 6 [12]. Similar absence of clear-cut correlation between base line inflammatory indices and response/remission rates at week 6 or week 54 were observed in the CD vedolizumab trial [14]. Strikingly, in a recent real-life cohort of 172 UC and CD patients treated by vedolizumab, an elevated CRP above normal value was the only predictor of lack of response to induction with vedolizumab [15]. Thus, more data is needed to support or refute the 'baseline CRP paradigm' derived from anti-TNF experience in the realm of the anti-integrin agents.

Thickened bowel wall and increased blood perfusion are typical features of inflammation and can be assessed by bowel ultrasound, which could be used as a simple and non-invasive technique for monitoring and optimizing the biological treatment. In a study of 24 consecutive patients receiving biological therapy, sonographic changes including reduction in the thickness of the bowel wall and Doppler blood flow were significantly more marked in Crohn’s patients who achieved clinical-biological response, compared to those patients who did not respond to treatment [16]. In another prospective longitudinal study of 30 patients receiving immunomodulators and/or biological treatment, 18 (60 %) patients exhibited endoscopic remission (CDEIS <6 points); of these patients, 15 (83.3 %) had normalized sonographic findings, with a good correlation between endoscopic remission and sonographic normalization (κ = 0.73, *p* < 0.001) [17]. Transmural healing evaluated by bowel ultrasound can be achieved in 17 of 66 patients with CD treated with anti-TNF-[alpha] agents and significantly correlates with MH.[18].

Fecal calprotectin (FC) has been shown to be useful in predicting relapse of quiescent IBD patients [19]. In a prospective study of UC patients on IFX, baseline FC was not associated with risk of relapse,whereas two consecutive FC > 300 mg/kg had 100 % specificity for relapse [20]. In another prospective study of UC patients in clinical and endoscopic remission, FC > 100 had 65 % of specificity and 88 % of NPV for relapse in 3 months, while FC > 250 had 85 % specificity, 88 % NPV for relapse in 3 months [21].

Overall, the majority of data supports ascertaining active inflammation and excluding other causes for symptoms as the most important objective measure to be implemented before induction with biologic therapies. Finer sub-groups distinction and even dichotomies may exist regarding the influence of different values (i.e. very high compared to high values) of inflammatory markers elevation on the outcome of therapy, and merit further studies. Additionally, sparse data indicates some value for week 2 CRP normalization in UC patients as associated with long-term response [22], but whether there is any role for routine monitoring of inflammatory and/or pharmacokinetic indices during the induction phase itself remains to be determined.

### After induction and during maintenance

It has been repeatedly shown that over half of the patient with CD in clinical remission may still have active inflammation as gauged by biochemical and/or endoscopic measures [23, 24].

Monitoring of patients in the immediate time-point after completion of induction seems to provide valuable clues as to the persistence of residual inflammation, and can be useful for identifying patients with high risk of unfavourable long-term outcome. For instance, normalization of inflammatory indices at week 10–14 of therapy, in the form of calprotectin normalization and/or CRP normalization or reduction, were found to predict long term outcomes including clinical remission and mucosal healing by most [25–29] but not all [30], studies. Along with reduction/normalization of inflammatory indices, infliximab levels higher than 3 to 4 mcg/ml at week 8 to 14 were also shown to predict long term response to maintenance therapy [25, 28, 31]. In a recent prospective observational study of 93 patients in clinical remission after infliximab induction, elevated CRP levels and infliximab drug levels below 5.5 mcg/ml were predictive of subsequent loss of response [32]. Thus, inflammatory and pharmacokinetic assessment at the end of induction and at early stage of maintenance phase may be justified in order to identify patients with high risk of therapy failure later on, in whom pre-emptive measures may be indicated.

Moreover, in studies of repeated evaluations of inflammatory markers in patients in remission, elevation of Calprotectin and CRP were shown to precede clinical flares by 3–6 months, thereby allowing to identify patients with imminent risk of disease relapse, at least in the short-medium time range [20]. Similarly, in the ULTRA 3 extension study of adalimumab in UC patients, both increasing CRP levels and decreasing albumin concentrations during treatment were identified as significant factors associated with a subsequent loss of remission [33]. Calprotectin was not evaluated in this study, but a metaanalysis of fecal calprotectin for predicting relapse in patients with quiescent IBD disclosed comparable ability of calprotectin to predict relapse in UC and CD patients, with an overall diagnostic area under the curve of the receiver-operaor curve of 0.83 [19]. Coupled with inflammatory markers and pharmacokinetic measurements mentioned above, evolution of immunogenicity, although also associated with reduced drug levels, was shown to independently predict future loss of response. In a prospective study of 125 IBD patients receiving infliximab, evolution of antibodies-to infliximab (ATI) preceded clinical loss of response in over 50 % of patients [34]. Similar results were reported with a cohort of adalimumab-treated patients, in whom 20 % developed anti-adalimumab antibodies which predicted future biochemical and clinical loss of response [35], and a two studies have found that anti-drug antibodies even in the presence of circulating infliximab levels, are associated with loss of response [36, 37].

Whether such inflammatory markers and/or pharmacokinetic and immunogenicity abnormalities which antedate clinical flares can be employed for actual change of management in patients in remission was hitherto investigated by three clinical trials. In the landmark TAXIT trial, stable patients under infliximab maintenance therapy (approximately 80 % were in complete clinical remission according to clinical scores) were first dose-optimized to achieve infliximab serum concentration between 3 to 7mcg/ml and then randomly allocated to continued conventional care or to therapeutic drug monitoring (TDM) -based dose-optimization strategy [38]. Although the pre-defined outcome of remission at week 52 was not different between the two arms, this was most likely due to study design allowing dose-adjustments in both arms until trial end-point. However, notably there was significant benefit for TDM-based arm as gauged by less clinical flares during the trial compared the standard treatment, indicating the superiority of the proactive approach over the reactive one in maintaining real enduring remission without disease relapses. A second trial examined UC patients in clinical remission yet with calprotectin levels above 50mcg/gr, and randomized them to continue same-dose mesalamine treatment or to escalate the dose of the 5-ASA [39]. This study found that escalating the dose of patients with clinical remission resulted in significant higher rate of biochemical remission (Calprotectin <50mcg/gr) at the end of the 6 weeks trial. Importantly, patients whose calprotectin levels were above 200mcg/ml had shorter relapse-free duration compared to those with calprotectin levels below this threshold [39]. Finally, a Swedish trial randomized UC patients in remission to an active arm of 5-ASA comprising of dose increase if calprotectin levels rose above 300mcg/ml versus a continued treatment arm, in which patients continued same-dose 5-ASA regardless of asymptomatic calprotectin elevations [40]. Only a numeric trend in favour of the active arm, but not a statistical significance difference, was found for the primary outcome of number of relapses in the entire cohort during the 18 month trial. However, the secondary outcome of number of relapses among UC patients in remission with calprotectin above 300mcg/ml has shown a significant reduction in clinical remission for the active arm, in which pre-emptive dose-escalation was instituted [40]. Taken together, the observational data about the correlation of elevated inflammatory markers and/or evolving pharmacokinetic derangement with soon-to-follow clinical flares, along with the above preliminary interventional data from clinical trials, suggest that the possible usefulness of a proactive approach whereby therapy may need to be modified in asymptomatic patients based on biochemical/pharmacokinetic monitoring. However, more controlled data is needed to define the best cut-off to define the abnormal values of the measured monitored parameters, the sub-populations who will benefit the most and the type of intervention which will be cost-effective.

### At loss of response

Similar to the situation at the initiation of therapy, the single most important factor to ascertain at the time of loss of response is the presence of active IBD inflammation as the cause for re-emerging symptoms. Indeed, myriad reasons may mimic a flare of IBD but will not be amenable to optimization of the IBD-directed therapy [41]. These include causes un-related to IBD, such as irritable bowel syndrome as well as aetiologies in-directly related to IBD (super-imposed infections, cancer) and even non-inflammatory causes directly related to the IBD (fibrotic stricture, bile-salt diarrhoea). Thus, astute management of patients with seeming loss of response start with obtaining objective measures to verify active inflammation is responsible for re-emerging symptoms, either by biochemical indices or endoscopic/imaging evidence. Once active IBD inflammation has been ascertained, TDM data can be very useful in guiding the therapeutic management. Thus, several observational studies have shown that levels of drugs and anti-drug antibodies correlate with response to common interventions for loss of response [42–44]. Specifically, patients with adequate drug levels and active inflammation will usually respond better to switch to another class of drugs. However, in selected cases, optimizing the anti-TNF class may sometimes still be warranted for individual patients who may require higher concentrations for response especially if they have exhausted other therapeutic modalities. Patients with low drug levels and low titer anti-drug antibodies will generally respond to dose-intensification, and patients with low drug levels and high anti-drug antibodies will generally require to in-class switch of biologic [41], or possibly to an addition of an immunomodulator in attempt to revert immunogenicity [45].

Notably, however, these algorithms are still imperfect, and were estimated to be able to guide therapy in 72 % of patients, whereas PK/immunogenicity results may come back in a grey-zone range for the remaining 28 % of patients for whom future studies are needed to refine TDM algorithms [44]. Nonetheless, it is important to note that a small randomized clinical trial, showed significant cost-savings of over 5000 euro for patients with loss of response to infliximab managed by TDM-based algorithm compared to standard dose-intensification protocol, with comparable clinical outcomes [46]. Thus, overall the role of TDM-guided therapy in patients with loss of response seems quite established, and several algorithms have been proposed to address this clinical scenario [47, 48], although more data is still needed to define the optimal thresholds for the different interventions and to reconcile inter-assay differences.

### Before and after stopping biologic therapy

A study of 52 patients from Finland, who stopped infliximab in deep remission after over one year of therapy, included a mixed population of UC and CD patients and could not identify factors predicting of relapse after therapy cessation [49]. In contrast, in the STORI trial whereby patients in clinical remission during combination therapy with infliximab + immunomodulators stopped infliximab therapy, elevation of calprotectin preceded and identified future flaring patients. Moreover, patients who discontinued infliximab while having clinical remission yet with elevated CRP had higher risk of relapse, probably indicating underlying asymptomatic inflammtion and lack of deep remission in this subgroup [50]. Interestingly, patients with low drug levels of infliximab, and even those with nil levels of infliximab and adalimumab, before stopping anti-TNF treatment during long-lasting remission, were found by two separate studies to sustain lower risk of relapse after anti-TNF cessation [50, 51]. This seemingly paradoxical finding indicates that a certain small subset of patients possibly exist, in whom biologic therapy was useful for induction of remission - deep enough to be maintained later-on independent of a pharmacokinetic/immunogenicity problem that later arose. Thus, incidental low/undetectable drug levels in patients with long-term deep remission may contra-intuitively identify patients whose remission is no longer dependent on anti-TNF therapy for its maintenance, and in whom the biologic may be discontinued. At any rate, although more data is needed, there seems to be a role for continued biochemical monitoring of patients who have stopped biologic treatment in remission, with the aim of early identification of patients at risk for relapse.

### Role of endoscopy and mucosal healing assessment for optimizing therapy

Throughout this review, we have focused on non-invasive objective measures for monitoring IBD patients. However, emerging data suggests that mucosal healing may be the ultimate therapeutic goal which may be strongly associated with alteration of disease natural history and reduction of future complications and surgeries [52–55]. As such, it is biologically reasonable that endoscopic assessment of mucosal healing will provide superior prediction power of future patient course compared to non-invasive blood and stool parameters. A comprehensive assessment of mucosal healing concept is beyond the scope of the present review. However, it is notable that in a paediatric prospective observational study of UC patients treated with infliximab, MH at week 8 was better correlated with one year corticosteroid remission compared to week 8 CRP level although not better than PUCAI clinical score for this purpose [56] and MH was also found to be the only factor predictive of future colectomy in a FENCH cohort of UC patients treated with infliximab [54]. Therefore, it is plausible to assume that mucosal healing, being more closely correlated with absence of bowel wall anatomic damage than surrogate biochemical indices of inflammation, will better serve to identify patients at risk for future relapses. In this respect, several studies recently have pointed to levels of anti-TNF drugs that are associated with higher rates of mucosal healing, rather than merely associate with clinical response [57, 58], and it is likely that in the future, our target concentrations towards which biologics dosing is adjusted will be governed by this ‘mucosal healing therapeutic drug window’.

In the randomised postoperative Crohn’s endoscopic recurrence (POCER) trial, 177 consecutive patients with Crohn’s disease undergoing intestinal resection of all macroscopic disease received standard prophylactic treatment with metronidazole and thiopurine, the later given to patients with high-risk features. Patients were then randomized into parallel groups: colonoscopy at 6 months (active care group, 122 patients) or no colonoscopy (standard care group, 52 patients). In the case of endoscopic recurrence (Rutgeerts score ≥ i2) at 6 months, patients in active group stepped-up to thiopurine, fortnightly adalimumab with thiopurine, or weekly adalimumab. The trial demonstrated that endoscopic recurrence at 18 months occurred in 60 (49 %) patients in the active care group and 35 (67 %) patients in the standard care group (*p* = 0•03). Complete mucosal normality was maintained in 27 (22 %) patients in the active care group versus four (8 %) in the standard care group (*p* = 0•03). This suggests that treatment according to clinical risk of recurrence coupled with early colonoscopy could play important role in optimizing post-operative treatment [59]

However, even endoscopic mucosal healing may not be the optimal marker, because careful studies have shown histologic inflammation to persist in a subset of patients with complete endoscopic healing of the mucosa [60]. Moreover, histologic inflammation even in the presence of complete mucosal healing was associated with future relapse, [61]. Interestingly, while calprotectin was found to correlate with mucosal healing in both CD and UC patients [62], a recent small study has found that in UC patients with clinical and endoscopic remission, low calprotectin was more useful to rule-out future relapses compared to normal histology score [63]. This illustrates the persisting challenges in defining the best outcomes in endoscopy and biochemical analysis that need to be jointly sought and combined for the purpose of optimal risk-stratification of IBD patients. Moreover, patient acceptability of repeated endoscopies is also a common obstacle, and more patient-friendly techniques for disease monitoring, especially for CD patients, may be required. Whether these will include capsule endoscopy and/or additional novel patient-friendly modalities remains to be seen.

## Conclusion

The plea for closer monitoring of IBD patients seems increasingly justified as our understanding evolves to acknowledge that patients’ clinical symptoms may reflect the tip of the iceberg of underlying incessant inflammation and consequent future bowel damage. Coupled with the non-negligible costs of therapy, and the over-all worse prognosis of moderate-severe patients who are the usual recipients of biologic therapies, this challenging patients seem to be the first candidates for this more proactive strategy combining inflammatory and pharmacokinetic monitoring of objective inflammatory and pharmacokinetic measures. More data is still desirable to better define the exact parameters to be followed and their optimal thresholds, and to delineate the optimal cost-effective interventions for these patients.

## Abbreviations

IBD: Inflammatory bowel disease
CD: Crohn’s disease
UC: Ulcerative colitis
ATI: Antibodies-to infliximab
TDM: Therapeutic drug monitoring
